# Low Salt Delivery Triggers Autocrine Release of Prostaglandin E2 From the Aldosterone-Sensitive Distal Nephron in Familial Hyperkalemic Hypertension Mice

**DOI:** 10.3389/fphys.2021.787323

**Published:** 2022-01-06

**Authors:** Ava M. Zapf, Paul R. Grimm, Lama Al-Qusairi, Eric Delpire, Paul A. Welling

**Affiliations:** ^1^Molecular Medicine, Graduate Program in Life Sciences, University of Maryland Medical School, Baltimore, MD, United States; ^2^Department of Medicine, Johns Hopkins University, Baltimore, MD, United States; ^3^Department of Anesthesiology, Vanderbilt University Medical School, Nashville, TN, United States; ^4^Department of Physiology, Johns Hopkins University, Baltimore, MD, United States

**Keywords:** distal convoluted tubule, ASDN, FHHt, prostaglandin E2, low salt

## Abstract

Aberrant activation of with-no-lysine kinase (WNK)-STE20/SPS1-related proline-alanine-rich protein kinase (SPAK) kinase signaling in the distal convoluted tubule (DCT) causes unbridled activation of the thiazide-sensitive sodium chloride cotransporter (NCC), leading to familial hyperkalemic hypertension (FHHt) in humans. Studies in FHHt mice engineered to constitutively activate SPAK specifically in the DCT (CA-SPAK mice) revealed maladaptive remodeling of the aldosterone sensitive distal nephron (ASDN), characterized by decrease in the potassium excretory channel, renal outer medullary potassium (ROMK), and epithelial sodium channel (ENaC), that contributes to the hyperkalemia. The mechanisms by which NCC activation in DCT promotes remodeling of connecting tubule (CNT) are unknown, but paracrine communication and reduced salt delivery to the ASDN have been suspected. Here, we explore the involvement of prostaglandin E2 (PGE2). We found that PGE2 and the terminal PGE2 synthase, mPGES1, are increased in kidney cortex of CA-SPAK mice, compared to control or SPAK KO mice. Hydrochlorothiazide (HCTZ) reduced PGE2 to control levels, indicating increased PGE2 synthesis is dependent on increased NCC activity. Immunolocalization studies revealed mPGES1 is selectively increased in the CNT of CA-SPAK mice, implicating low salt-delivery to ASDN as the trigger. Salt titration studies in an *in vitro* ASDN cell model, mouse CCD cell (mCCD-CL1), confirmed PGE2 synthesis is activated by low salt, and revealed that response is paralleled by induction of mPGES1 gene expression. Finally, inhibition of the PGE2 receptor, EP1, in CA-SPAK mice partially restored potassium homeostasis as it partially rescued ROMK protein abundance, but not ENaC. Together, these data indicate low sodium delivery to the ASDN activates PGE2 synthesis and this inhibits ROMK through autocrine activation of the EP1 receptor. These findings provide new insights into the mechanism by which activation of sodium transport in the DCT causes remodeling of the ASDN.

## Introduction

Potassium homeostasis is dependent on ion transport processes in two adjacent distal tubule segments, the distal convoluted tubule (DCT), and the aldosterone-sensitive distal nephron (ASDN; Hadchouel et al., 2016; Welling, 2016). The transport activities of these different tubules are precisely regulated to either reabsorb sodium with chloride or exchange sodium with potassium, depending on physiological needs. Cells in the ASDN are the primary site of potassium secretion; sodium absorption through the apical Na^+^ channel (ENaC) creates a favorable driving force for potassium to be transported into the tubule lumen through the chief potassium excretory channels, renal outer medullary potassium (ROMK) and BK (Giebisch and Wang, 2010; Welling, 2016). The upstream DCT segment, which absorbs sodium chloride, influences potassium secretion, in part, by tailoring sodium delivery to the ASDN to match physiological demands for potassium secretion.

Distal convoluted tubule cells sense small physiological changes in plasma potassium levels and signal the change through a with-no-lysine kinase (WNK)-STE20/SPS1-related proline-alanine-rich protein kinase (SPAK) signaling network, called the “potassium switch” to adjust the activity of the thiazide sensitive sodium chloride co-transporter (NCC) (Hadchouel et al., 2016). In states of dietary potassium deficiency, the kinase cascade phospho-activates NCC to increase sodium reabsorption, decreasing distal sodium delivery to limit potassium secretion in the ASDN (Terker et al., 2015; Wade et al., 2015). When dietary potassium levels increase, activity of the kinase cascade and NCC diminish (Vallon et al., 2009; Rengarajan et al., 2014; Penton et al., 2016), increasing sodium delivery to increase electrogenic sodium-potassium exchange and urinary potassium excretion. Gain of function mutations in the with-no-lysine kinases, WNK1 and WNK4, cause Gordon’s familial hyperkalemia hypertension (FHHt), characterized by thiazide-treatable hypertension, hyperkalemia, hyperchloremia, and metabolic acidosis (Wilson et al., 2001). Loss-of-function mutations in the Kelch-like 3/Cullin3 ubiquitin E3 ligase complex, which targets the WNK kinases for proteasomal degradation also cause FHHt (Boyden et al., 2012; Louis-Dit-Picard et al., 2012).

In addition, limiting sodium delivery to the ASDN, switch activation in the DCT suppresses potassium secretion by triggering a distal nephron remodeling response (Lalioti et al., 2006; Grimm et al., 2017). We found that DCT-specific knockin of a constitutively activated SPAK kinase (CA-SPAK) is sufficient to drive the FHHt phenotype in mice (Grimm et al., 2017). Remarkably, genetic activation of NCC in the CA-SPAK was sufficient to induce a distal tubule remodeling response, characterized by DCT hypertrophy, atrophy of the connecting tubule (CNT), and a decrease in the ROMK and epithelial sodium channel (ENaC). Hydrochlorothiazide (HCTZ) reversed the changes in the CNT, indicating that activation of NCC is sufficient to drive remodeling of the downstream segment. The observation raises the possibility that the structure and function of the CNT is controlled by chemical signals that emanate from the DCT. Here, we consider the involvement of the local autocrine/paracrine, prostaglandin E2 (PGE2), which is well-known to inhibit the reabsorption of water (Orloff et al., 1965; Grantham and Orloff, 1968) and sodium (Stokes and Kokko, 1977) and the secretion of potassium (Jin et al., 2007) in the ASDN, although these effects can be species (Schafer, 2002) and receptor type dependent (Hebert et al., 1993; Olesen et al., 2016). Moreover, consumption of potassium-free diet, which activates NCC, also increases renal PGE2 levels, and inhibits ROMK (Jin et al., 2007). Collectively, these data led us to the hypothesis that activation of NCC causes an increase in PGE2 synthesis, which activates an inhibitory mechanism within the ASDN, resulting in a decrease in ROMK. To test this idea, we took advantage of the CA-SPAK mouse model to assess the dependence of PGE2 synthesis on NCC activation. We found that the NCC-dependent decrease in distal sodium delivery triggers PGE2 synthesis in and release from the connecting tubule, driving autocrine inhibition of potassium excretion *via* the EP1 receptor.

## Materials and Methods

### Animal Experiments

#### Mice and Dietary Manipulation

Experiments outlined were approved by the Johns Hopkins University Animal Care and Use Committee or The University of Maryland School of Medicine Institutional Animal Care and Use Committee. Control, CA-SPAK, SPAK KO, and C57BL/6 J wild-type mice (WT) purchased from Jackson laboratories, were housed in a temperature-controlled facility with a 12:12 h light:dark cycle within Johns Hopkins University or University of Maryland School of Medicine. Food and water were available *ad libitum*. Animals aged 8–14 weeks were acclimated to a control diet [1% K^+^, 0.3% Na^+^, and 0.9% Cl^−^, Teklad Diet (TD) 88238] for at least 7 days prior to beginning the experiment. In the studies involving control, CA-SPAK, or SPAK KO mice remained on the control diet for the duration of the study.

#### Metabolic Cage Studies and Blood and Urine Measurements

Mice were acclimated to metabolic cages (Nalgene) for 3 days prior to experimental manipulation. Urine output, food and water intake was monitored and measured daily. For tissue and blood collection, mice were anesthetized by intraperitoneal injection with ketamine-xylazine (100 mg/kg ketamine and 10 mg/kg xylazine). Blood samples were collected from the right carotid artery using a 1,000 μl pipette tip containing heparin and evaluated immediately for electrolyte analysis. Blood chemistry and gases (Na^+^, K^+^, Cl^−^, HCO_3_^−^, pH, hematocrit, and BUN) were measured using a 100 μl aliquot of whole blood using an i-STAT EC3 or EC8+ cartridge and i-STAT1 Handheld Analyzer (Abaxis). Remaining blood was immediately spun down to separate the plasma and frozen for later analysis. Urine was collected daily in 24-h increments and immediately stored at −80°C for later analysis of K^+^, Na^+^, and Cl^−^ excretion (CareLyte Chemistry Analyzer, Diamond Diagnostics) or measuring PGE2 levels. Although there were no differences in food consumption across all treatment groups, individual variability within groups was noted. To account for this, electrolyte excretion data were individually normalized to dietary intake. Plasma and urine osmolarity were measured using a vapor pressure osmometer (Wescor-Vapro 5520).

### Drug Treatments

Animals were assigned at random to receive either vehicle or drug treatment. Dose, administration of the drug and duration of the study are listed below in Table 1.

**Table 1:** Experimental design and drug treatments.

Experiment	Genotype used	Vehicle	Drug	Dose/Delivery	Length of study
HCTZ	Control, CA-SPAK, and SPAK KO	35% DMSO diluted in 0.9% saline	Hydrochlorothiazide (HCTZ) Sigma	25 mg/kg IP	4 days
EP1 antagonist	CA-SPAK	0.9% saline	EP1 antagonist Cayman Chemical SC-51089	6.5 mg/kg IP	4 days

### Western Blot Analysis

Tissue dissection, storage and preparation for western blots (WBs) were performed as previously described (Al-Qusairi et al., 2021). Briefly, cortex and medulla tissues were snap frozen in liquid nitrogen and then stored at −80°C until use. Frozen tissue was minced in HEENG buffer [20 mM Hepes (pH 7.6), 125 mM NaCl, 1 mM EDTA, 1 mM EGTA, 10% glycerol, 1% Triton X, and 0.5% SDS plus protein and phosphatase inhibitor] as previously described (Grimm et al., 2017). For reliable solubilization and resolution of membrane proteins, lysate buffer [20 mM HEPES, 125 mM NaCl, 1 mM EDTA, 1 mM EGTA, 10% glycerol, pH 7.5, plus 20% Triton-X, 10% SDS, protease (P8340, Sigma) and phosphatase inhibitor], was used. Samples were sonicated 2–3 times using 7 s pulses (20 s between pulses), then allowed to sit at room temperature for 15 min. The sample was then rotated at 4°C for 1 h followed by being centrifuged at max speed (~13,000 rpm) for 15 min at 4°C. The supernatant was collected and protein concentration was measured using Pierce BCA protein assay (23225, Thermo Scientific). Equal amount of protein (30–40 μg) was incubated in loading buffer containing 15% beta-mercaptoethanol for 30 min at room temperature prior to proteins being separated on 4–20 or 4–15% Mini or Midi-Protean TGX or Stain-free gels (Bio-Rad), and transferred to nitrocellulose membranes using Trans-Blot Turbo RTA Transfer Kit (170–4,270, mini or 170–4,271, midi, Bio-Rad). Membranes were blocked in 5% nonfat dry milk dissolved in Tris-buffered saline with 1% Tween 20 (TBS-T) 1 h at room temperature and incubated overnight at 4°C with primary antibodies (Table 2). Membranes were then washed in TBS-T three times for a total of 1 h at room temperature, then incubated for 1 h in TBS-T with horseradish peroxidase-conjugated secondary antibody, goat anti-rabbit IgG (1:5,000, 111-035-144, Jackson Immunoresearch), or goat anti-mouse IgG (1:5,000, 115-035-166, Jackson Immunoresearch). For normalization, Protean TGX gels were stripped and reprobed with either b-tubulin, b-actin, or GAPDH. Total protein was used for normalization when Stain-free gels were used. The same gel type (TGX or stain-free) was used for all proteins analyzed from the same study.

**Table 2:** Antibody list.

Antibody	Source	Species	Dilution and application (IF, immunofluorescence; WB, western blot)
Cox1	Cell signaling #4841	Rabbit	WB (tissue) 1:1,000 WB (cells) 1:2,000
Cox2	Abcam (ERP12012) Ab179800	Rabbit	WB 1:1,000
mPGES1	Cayman 160140	Rabbit	WB 1:1,000 IF 1:200
ENaC alpha	Johannes Loffing (Zurich University)	Rabbit	WB 1:500
ENaC gamma	Stressmarq SPC-405D	Rabbit	WB 1:3,000
Bk-beta1	Alomone APC-036	Rabbit	WB 1:6,000

In agreement with others, we found immunodetectable Cox2 runs as several distinct molecular bands (Sevigny et al., 2006; Raman et al., 2018), reflecting different glycosylated forms. In the kidney, we found two glycosylated forms of Cox2 at 72 kDa and ~66 kDa. For quantification, we measured both bands, and combined them as total Cox2.

### RNA Isolation, cDNA Synthesis, and qRT-PCR

Kidney cortical tissue was manually dissected, immediately placed in a 1.5 ml vial containing 300 μl of RNALater (Qiagen) and kept at −80°C until RNA extraction. RNA was isolated from the cortex using a Trizol/RNeasy hybrid protocol. Cortical tissue was placed in a BeadBug Eppendorf tube (D1032-15, Sigma) containing Trizol (1 ml per 0.1 g of tissue) and homogenized for 30 s at 3,000 rpm twice. Homogenate was transferred to a new Eppendorf tube and centrifuged at 12,000 *g* for 10 min 4°C to remove cellular debris. Chloroform was added (0.2 ml chloroform per ml of Trizol) and the sample was shaken vigorously, then allowed to sit at room temperature for 3 min. The sample was centrifuged at 10,000 *g* for 18 min at 4°C for phase separation. An equal volume of ethanol was added to the supernatant (aqueous phase), then the RNA was recovered using a RNeasy column (74106, Qiagen). RNA concentration and integrity were measured using NanoDrop Lite, and only samples with an A260/A280 value of 1.8–2.0 were used. cDNA synthesis was performed using SuperScript III Reverse Transcriptase (18080-51, Invitrogen). Primer sequences of Cox1, Cox2, mPGES1, mPGES2, cPGES, and EP1-4 are shown in Table 3 and were generated using MacVector version 8. Samples were denatured for 1 min at 95°C, followed by 95°C for 30 s, 52°C for 30 s, and 72°C for 30 s repeated for 35 cycles. Final extension time was 95°C for 15 s. qPCR was performed using Applied Biosystems with PowerUp SYBR Green (A25742). Changes in RNA abundance were quantified by accessing the cycle threshold (Ct) and using the 2^-ΔΔCt^ method (Livak and Schmittgen, 2001), with β-actin Ct values for normalization. All reactions were performed in triplicate for each animal and averaged.

**Table 3:** Primer sequences.

Gene	Forward primer	Reverse primer
Cox1	TACTCACAGTGCGGTCCAAC	GGGCCAGAAGCTGAACATCT
Cox2	ATTACTGCTGAAGCCCACCC	CATGGGAGTTGGGCAGTCAT
mPGES1	CAGATGAGGCTGCGGAAGAA	TATCCAGGCGATCAGAGGGT

### Immunolocalization and Quantification of mPGES1

Anesthetized mice were fixed by perfusion through the left ventricle of 2% paraformaldehyde in PBS 5 min at room temperature after initial perfusion with cold PBS for 2 min. The kidneys were removed and kept in 2% paraformaldehyde at 4°C for 24 h. The kidneys were rinsed with PBS and embedded in paraffin. Cross-sections of 5 μm thickness were picked up on poly-L-lysine coated glass coverslips and dried on a warming plate. Sections were deparaffinized with xylene 2x for 2 min each, followed by a graded ethanol series (100, 90, 70, 50, 35, and 0% ethanol) to distilled water to remove SDS. Epitope retrieval was conducted by placing the coverslips in retrieval buffer (1 mM Tris, 0.5 mM EDTA, and 0.02% SDS), and pH adjusted to 8.0. The retrieval solution was heated to boiling in a microwave (~5 min), transferred to a conventional boiling water bath for 15 min, and then allowed to cool to room temperature before sections were washed with distilled water to remove SDS. Sections were briefly allowed to dry and a liquid barrier drawn around the sample with a PAP pen. Sections were blocked for 20 min with Image-iT FX (Invitrogen), washed twice with PBS, and then blocked with homemade blocking solution (50 ml 1xPBS, 1% BSA, 50 mM glycine, 0.2% sodium azide, pH 7.2) for 30 min at room temperature. Primary antibodies were prepared in incubation medium (PBS, 1% BSA, 1% Tween 20, 0.35 M NaCl, and 0.2% sodium azide), kept in a moist chamber overnight at 4°C. After sections were washed for 1 h, Alexa Fluor secondary antibodies were diluted in incubation medium and applied to the sections for 1 h at room temperature. Sections were washed with high-salt wash buffer (three times) for 1 h, then mounted on slides in Vectashield mounting media (Vector Laboratories).

Image acquisition was performed with a Zeiss LSM 700 confocal microscope, using the same laser power, pinhole, and acquisition settings. Specific distal tubule segments were identified using a combination of distal tubule specific markers. The early DCT segment (DCT1) was defined as the expressing NCC, but no calbindin; the late DCT (DCT2) expressed both NCC and calbindin; the CNT expressed calbindin, but no NCC and weaker AQP2 than the CCD; and the CCD expressed strong AQP2, but no calbindin. For each individual animal, the average fluorescence signal intensity of mPGES1 was measured using Velocity Image Analysis Software. mPGES1 levels in the cytoplasm of individual cells (5–10 cells per tubule segment), were measured, using a free-hand tracing tool to exclude the nucleus and capture the entire cytoplasmic area as the region of interest. A total of 8–10 tubules per distal tubule segment (DCT1, DCT2, CNT, and CCD) were used for quantification for each animal. The mean signal intensity for the 8–10 tubules quantified were averaged for each animal (four control and four CA-SPAK) and are represented as individual points. To limit variability in mPGES1 quantification, immunofluorescence (IF) was performed in groups of two or three control and CA-SPAK sections and imaged on the same day.

### mCCD-CL1 Cell Experiments

Mouse CCD cells (mCCD-CL1) were cultured as previously described (Gaeggeler et al., 2005; Fodstad et al., 2009). Cells were routinely passaged and maintained in T75 flasks at 37°C in a 5% CO_2_ incubator in DMEM/F12 media supplemented with [EGF 10 ng/ml, T3 1nM, dexamethasone 50 nM, Insulin-Transferrin-Selenium (ITS-G) 1x, penicillin–streptomycin, and 2% FBS]. For experimental procedures, Costar Transwell polycarbonate inserts (#3401) were coated with rat tail collagen prior to cell seeding. Cells were seeded at a 1x cell density onto the rat tail collagen coated wells and grown in growth media for 4 days before changing the media to filter media (lacking EGF and reduced dexamethasone). Transepithelial resistance measurements were continuously checked until the resistance of 3,000–4,000 ohms*cm^2^ was reached, denoting confluence (Gaeggeler et al., 2005). Once confluence was reached, the basolateral media was replaced with plain DMEM/F12 (nothing added) and apical media was changed to homemade DMEM/F12 with varying concentrations of sodium chloride added (0, 20, 40, 60, or 80 mM NaCl). Cells were incubated for 24 h before apical and basolateral media was collected for later PGE2 ELISA analysis. Cells were harvested by first washing cells 2x with cold PBS, then added RIPA buffer or Trizol directly to the transwell, for protein or RNA extraction, respectively. Cell protein lysate was rotated for 1 h at 4°C, then centrifuged full speed for 10 min at 4°C. Supernatant was transferred to a clean Eppendorf tube and protein concentration was measured using BCA Bradford method. Cells in Trizol were immediately transferred into a RNeasy column for isolation.

### Prostaglandin E2 ELISA Measurements

Kidney tissue was snap frozen at −80°C until protein lysates were made. The kidney cortex from one-half of one kidney (~30–50 mg) was sonicated in homogenization buffer 1 ml/100 mg of tissue (1xPBS, pH 7.4, 1 mM EDTA, and 10μM indomethacin). PGE2 was extracted with 100% ethanol, using four times the sample volume. Samples were incubated on ice for 5 min at 4°C, then centrifuged at 3,000 *g* for 10 min at 4°C to remove precipitated proteins. Supernatant was transferred to a clean test tube, then ethanol was evaporated using a Savant SpeedVac 100. The sample was resuspended in 500 μl ELISA buffer and vortexed. A standard curve of PGE2 was prepared per manufactures protocol (Cayman Chemical 514010), and each sample (50 μl/well) was assayed at least in duplicate. Standard curve of PGE2 and concentration of PGE2 of the samples was determined using the analysis template provided by Cayman Chemical. Samples were normalized to the tissue starting weight.

Urine samples were kept at −80°C until assayed. To remove proteins and cellular debris from the urine, samples were spun at max speed (~13,000 rpm) for 10 min at 4°C. Samples were then assayed in at least duplicates. Total PGE2 levels were determined by multiplying the concentration of each sample by the 24-h total urine volume.

### Statistical Analysis

Data are represented as ± SEM. Statistical analysis was performed using GraphPad PRISM version 8. When single dependent variables were compared, data was analyzed using unpaired two-tailed Students *t*-test. When comparing multiple-groups with a single dependent variable and variable SD, statistical significance was determined using Welch one-way ANOVA and Brown-Forsythe tests. Comparison of two different variables (e.g., genotype and drug) and a dependent variable (PGE2 levels) was assessed using two-way ANOVA. Tukey’s Multiple Comparison test was used for *post hoc* analysis. Comparisons were considered significant when *p* ≤ 0.05.

## Results

### NCC-Dependent PGE2 Synthesis

**Figure 1:**
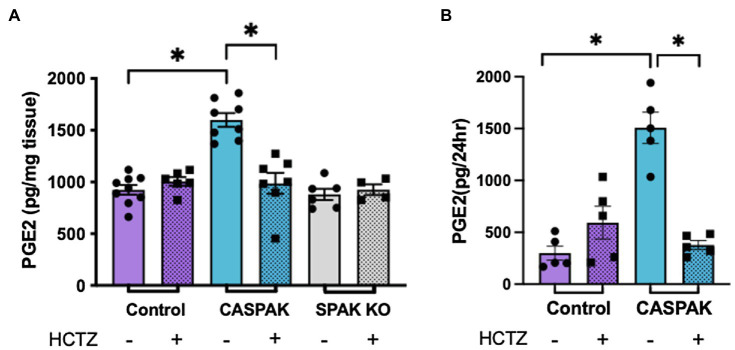
Activity of sodium chloride cotransporter (NCC) drives increased prostaglandin E2 (PGE2) synthesis. PGE2 levels after 4 days of treatment with hydrochlorothiazide (HCTZ) or vehicle. **(A)** Cortical tissue and **(B)** Urine, in control, CA-SPAK and SPAK KO mice. Statistical significance evaluated by two-way ANOVA (see text), followed by Tukey’s *post hoc* tests (^*^p < 0.05).

Aldosterone sensitive distal nephron remodeling in the DCT-specific CA-SPAK mice is manifested by a decrease in ROMK and ENaC protein abundance, and by a structural atrophy of the CNT (Grimm et al., 2017). This response is reversed by inhibiting NCC with HCTZ, suggesting a chemical communication between the DCT and ASDN. To determine whether PGE2 is a mediator, PGE2 levels were measured by ELISA in control, CA-SPAK and SPAK KO mice treated with vehicle or HCTZ for 4 days, and the results are shown in Figure 1.

A two-way ANOVA was performed to analyze the effect of genotype (Control, CA-SPAK, and SPAK KO) and treatment (vehicle, HCTZ) on PGE2 levels in cortical tissue (Figure 1A). We found a significant interaction between genotype and HCTZ treatment, *F*(2,34) = 18.1, *p* < 0.007. Simple main effects analysis revealed genotype has a significant effect on PGE2 in kidney cortical tissue, *F*(2,34) = 19.84, *p* < 10^−4^. CA-SPAK mice have significantly higher levels of PGE2 compared to control or SPAK KO mice (*p* < 10^−4^, Tukey multiple comparison; Figure 1A). HCTZ treatment significantly lowered PGE2, *F*(1,34) = 8.3, *p* < 10^−4^, to control levels in CA-SPAK mice (*p* < 10^−4^, Tukey multiple comparison) but had no effect on PGE2 in control and SPAK KO mice (Figure 1A). In summary, CA-SPAK mice have higher levels of PGE2 in the kidney cortex and PGE2 levels are uniquely sensitive to HCTZ.

Urinary PGE2 levels paralleled PGE2 levels in kidney tissue (Figure 1B). Like in tissue, a significant interaction between genotype and treatment, *F*(1,16) = 37.4, *p* < 10^−4^, was observed. Simple main effects analysis revealed urinary PGE2 was significantly increased, *F*(1,16) = 18.2, *p* = 0.0006, in CA-SPAK mice compared to control mice (*p* = 0.015) and was HCTZ sensitive, *F*(1,16) = 12.9; *p* < 0.002, in CA-SPAK mice (*p* < 10^−4^) but not control (Figure 1B). Collectively, the results indicate constitutive activation of NCC causes an increase in kidney PGE2.

**Figure 2:**
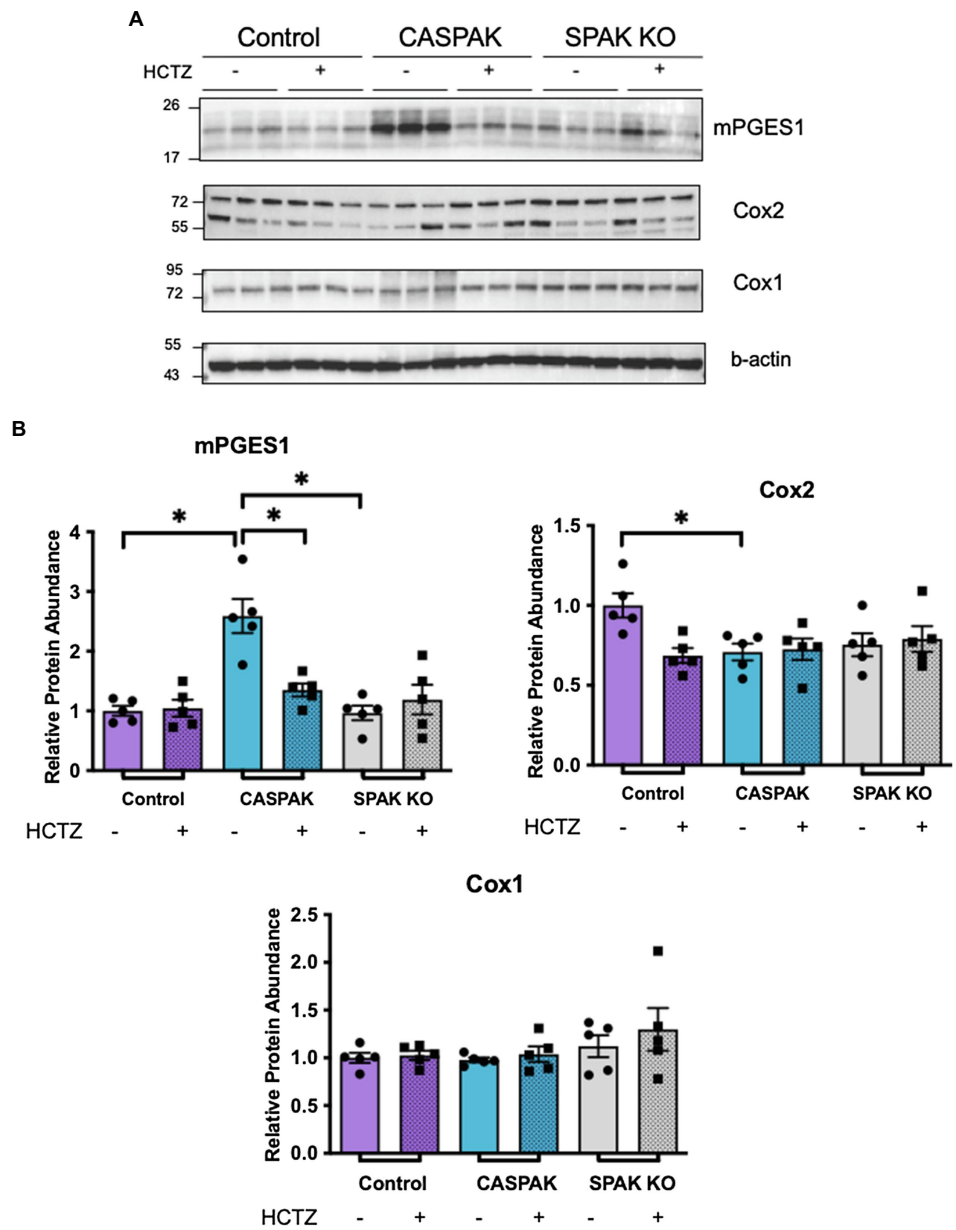
Increased mPGES1 protein abundance is dependent upon activation of NCC. Western blot (WB) analysis of PGE2 synthesis enzymes in tissue of kidney cortex. **(A)** mPGES1, total Cox2 (both bands), and Cox1 **(B)** quantification. Statistical significance evaluated by two-way ANOVA (see text), followed by Tukey’s *post hoc* tests (^*^p < 0.05).

To determine if the response is dictated by induction of PGE2 synthesis enzymes, protein abundance of Cox1, Cox2, and mPGES1 was evaluated by WB analysis in the kidney cortex (Figure 2). Two-way ANOVA analyses were performed to evaluate the effect of genotype (Control, CA-SPAK, and SPAK KO) and treatment (vehicle, HCTZ) on PGE2 synthesis enzymes. Neither genotype nor treatment had significant effects on Cox1. With Cox2, there was a significant interaction between genotype and treatment, *F*(2,24) = 4.32, *p* = 0.025. Multiple comparison analysis revealed Cox2 is slightly more abundant in control mice than CA-SPAK or SPAK KO mice and is HCTZ sensitive (*p* < 0.03). The most robust differences were observed with mPGES1. A significant interaction between genotype and treatment was observed with mPGES1, *F*(2,24) = 9.722, *p* = 0.0008. Simple main effect analysis revealed genotype, *F*(2,24) = 17.22, *p* < 10^−4^, and treatment, *F*(1,24) = 4.804, *p* = 0.038, had a significant effect on mPGES1 protein abundance. CASPAK mice have significantly higher levels of mPGES1 compared to control or SPAK KO mice (*p* < 10^−4^, Tukey multiple comparison) and was HCTZ sensitive (*p* = 0.0008, Tukey multiple comparison; Figure 2). HCTZ decreased mPGES1 levels in CA-SPAK mice to control levels within 4 days but had no effect on basal mPGES1 levels in control mice (Figure 2). In summary, these data indicate that constitutive activation of NCC selectively drives mPGES1, coinciding with an NCC-dependent increase in tissue and urinary PGE2 levels.

### NCC Drives PGE2 Synthesis in the CNT

**Figure 3:**
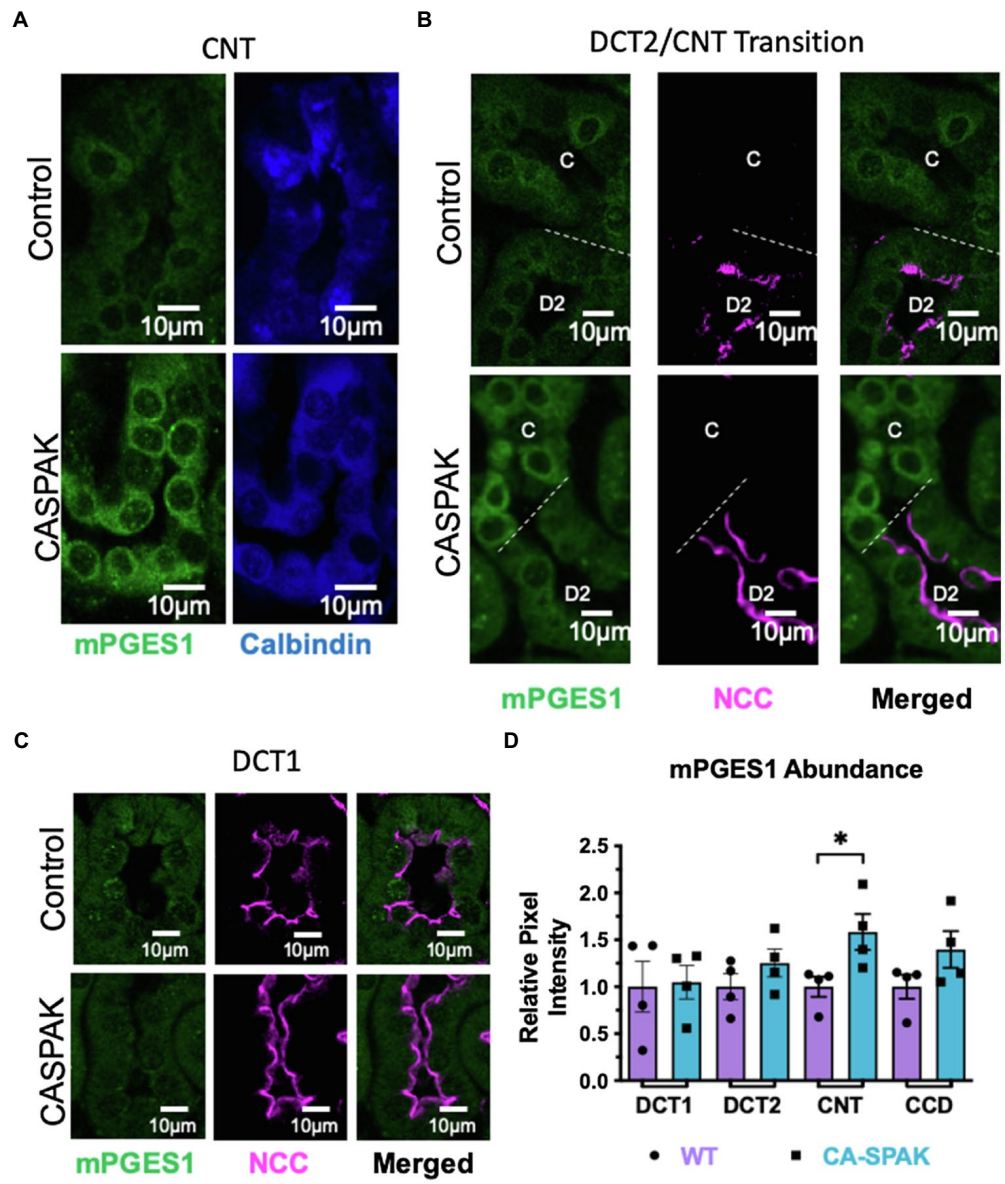
mPGES1 localization is increased exclusively in the connecting tubule (CNT). Control and CASPAK mPGES1 localization in **(A)** CNT only, **(B)** DCT2 (D2) to CNT, **(C)** transition segment and **(C)** DCT1, **(D)** Quantification. Tubule segments were identified by colabeling with specific antibodies (DCT1: NCC+ calbindin-; DCT2: NCC+ calbindin+; CNT: calbindin+ AQP2−/+; CCD: calbindin- AQP2+). ^*^p < 0.05; statistical significance evaluated by two-tailed *t*-test.

Semi-quantitative immuno-microscopy studies were performed to determine the cellular site(s) of mPGES1 induction. For these studies, mPGES1 was colocalized with distal tubule-markers and cytoplasmic mPGES1 pixel intensity was quantified in the individual distal nephron segments. Surprisingly, we found mPGES1 protein abundance was increased in the CNT rather than the DCT (Figures 3A–D). The data suggest that constitutive activation of NCC indirectly activates PGE2 synthesis selectively in the CNT.

### Salt-Dependent PGE2 Synthesis

**Figure 4:**
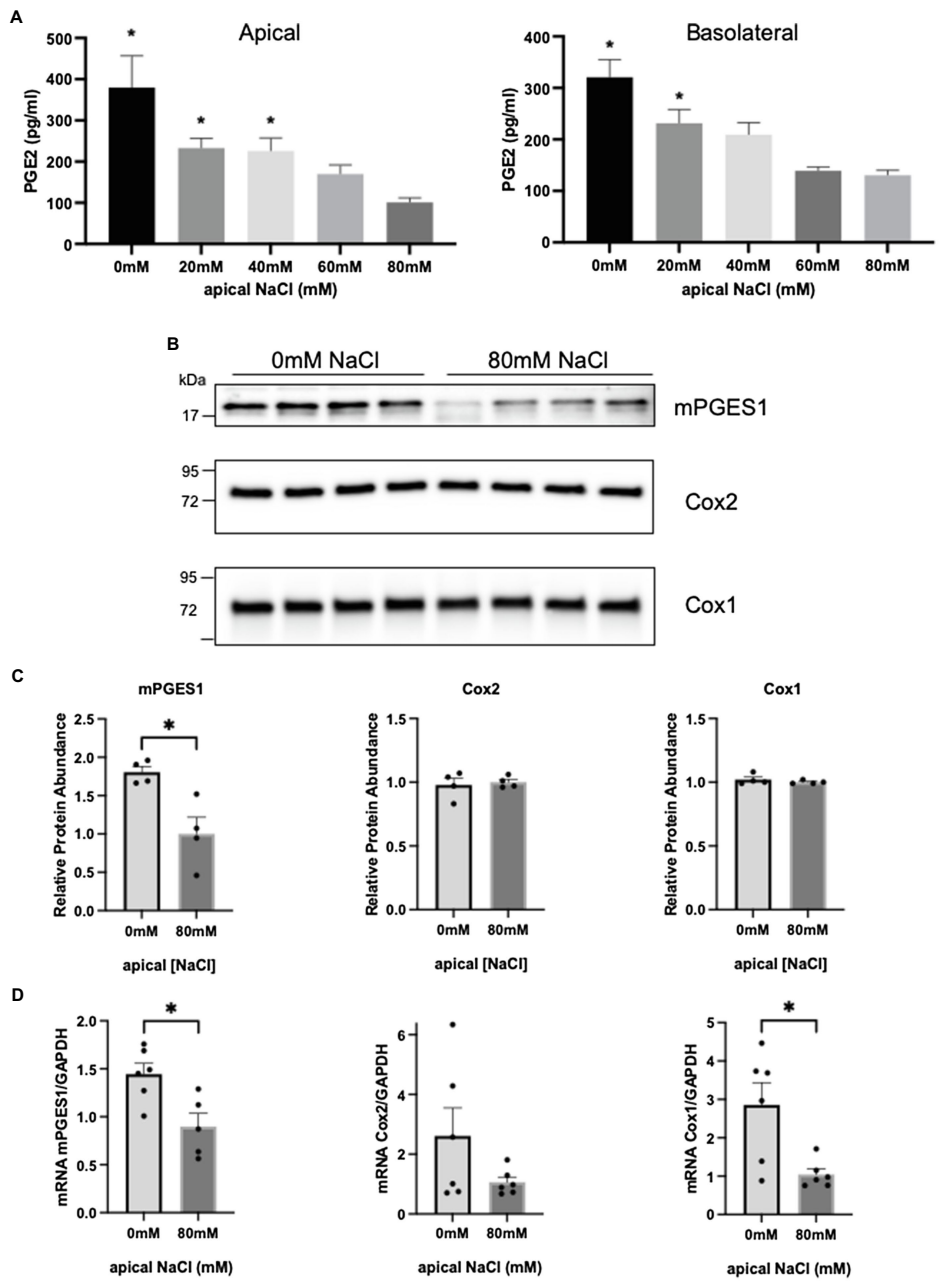
Prostaglandin E2 increases when apical NaCl decreases to a minimum of 40 mM in a mPGES1 dependent manner in mouse CCD cell (mCCD-CL1). Each experimental condition was compared to 80 mM NaCl. **(A)** PGE2 synthesis levels after 24 h stimulation of various apical NaCl concentrations in the apical compartment (left) and basolateral compartment (right). **(B)** WB analysis of PGE2 synthesis enzymes from same mCCD-CL1 cells. **(C)** Quantification of the western blots shown in **B**. **(D)** mRNA abundance of PGE2 synthesis enzymes normalized to GAPDH. ^*^p < 0.05; Statistical significance in **A** was evaluated by Brown-Forsythe and Welch ANOVA tests and **B–D** with two-tailed *t*-test.

We wondered if a decrease in salt (sodium chloride) delivery to the CNT might be the stimulus for driving PGE2 synthesis in the ASDN when NCC is activated, similar to the way low chloride stimulates PGE2 synthesis and release from macula densa cells (Yang et al., 2000; Peti-Peterdi et al., 2003) by activation of Cox2 (Harris and Breyer, 2001). To test this, we measured PGE2 release from mCCD-CL1 aldosterone-sensitive principal cells *in vitro* (Gaeggeler et al., 2005), and over a titration of NaCl concentrations. For these studies, cells were grown to confluence (transepithelial resistance >4,000 ohms-cm^2^) on permeable transwell inserts, and the sodium-chloride concentration was varied in the apical compartment, mimicking changes in sodium delivery (Good and Wright, 1979; Good et al., 1984). PGE2 was measured in the apical and basolateral media 24 h after changing the sodium-chloride concentration. As shown in Figure 4A, a significant increase in PGE2 was detected in apical and basolateral compartments when NaCl was decreased from 80 to 40 mM, and PGE2 continued to increase as apical NaCl concentration was decreased from 40 to 0 mM (Figure 4A).

### Salt-Dependent Activation of mPGES1

Immunoblotting revealed that mPGES1 protein abundance was significantly increased when the apical sodium-chloride concentration was reduced (Figures 4B,C), similar to activating NCC *in vivo*. The increase in mPGES1 protein was paralleled by an increase in transcript, suggesting reduced salt delivery stimulates PGE2 synthesis by activating mPGES1 gene expression (Figure 4D). Although, an increase in Cox1 mRNA abundance was also observed, it was not paralleled by an increase in Cox1 protein abundance (Figure 4D).

### Autocrine Release of PGE2 Inhibits ROMK *via* the EP1 Receptor

**Figure 5:**
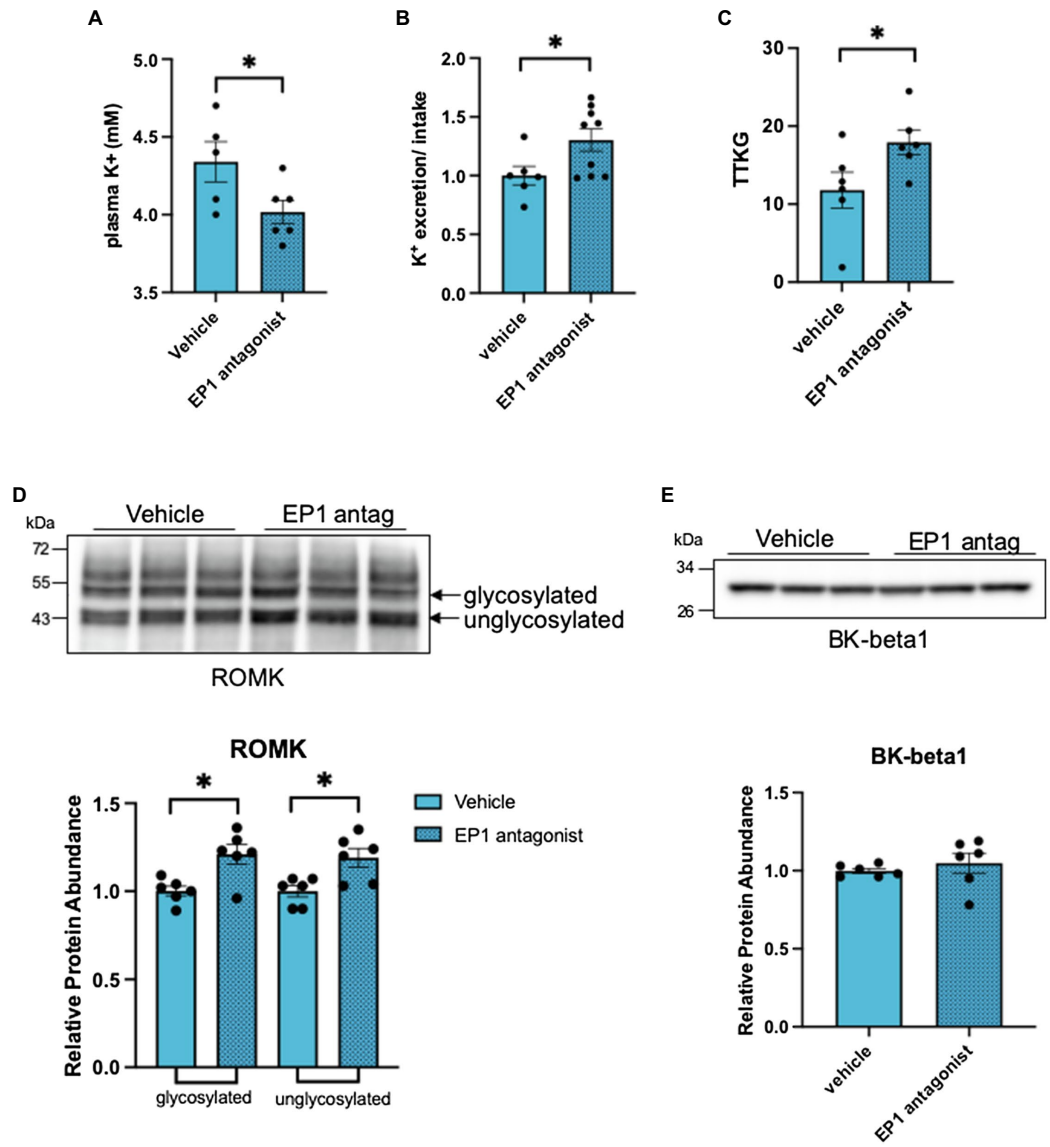
Inhibition of the EP1 receptor rescues renal outer medullary potassium (ROMK) protein abundance. **(A)** plasma K+, **(B)** Total K+ excretion: total K+ intake ratio per individual mouse, **(C)** transtubular K+ gradient of an individual mouse, and **(D)** western blot and analysis of ROMK and **(E)** BK-beta1. ^*^p < 0.05; statistical significance evaluated by two-tailed *t*-test.

**Figure 6:**
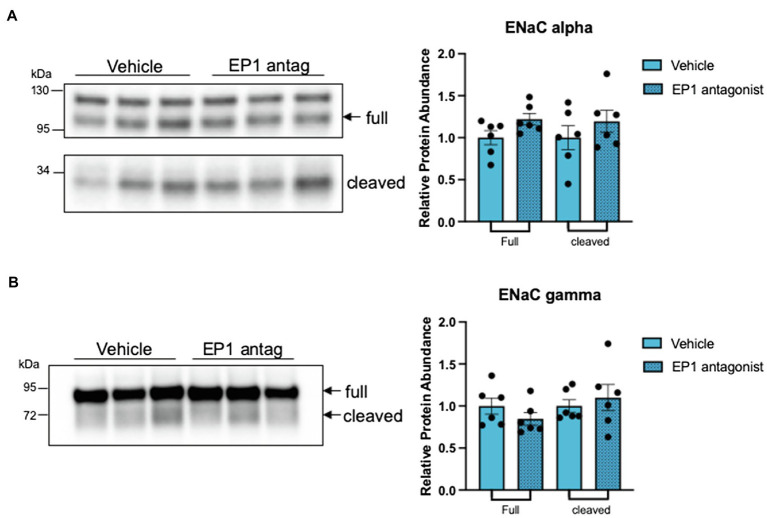
EP1 inhibition does not change expression of full length or cleaved forms of epithelial sodium channel (ENaC). Western blot and quantification of full length and cleaved forms of **(A)** ENaC alpha (arrows point to ENaC specific bands that were quantified) and **(B)** ENaC gamma. ^*^p < 0.05; statistical significance evaluated by two-tailed *t*-test.

The observations above raise the possibility that PGE2 acts as an autocrine mediator of the ASDN remodeling response when salt delivery to the segment diminishes. Because the decrease in ROMK is central to the hyperkalemic phenotype in CA-SPAK mice (Grimm et al., 2017), and PGE2 inhibits ROMK *via* the EP1 receptor in dietary potassium restriction (Jin et al., 2007), we explored the ROMK remodeling response in CA-SPAK mice. For these studies, CA-SPAK mice were treated with vehicle or the EP1 antagonist SC-51089 for 4 days. As shown in Figure 5, SC-51089 restored plasma K^+^ in the CA-SPAK mice to control levels (3.9 mM) previously reported for control mice (Grimm et al., 2017). Measurements of total K^+^ excretion normalized to total K^+^ consumed revealed a significant increase in overall urinary potassium excretion in SC-51089 treated mice compared to vehicle, consistent with the significantly increased potassium secretion from the ASDN as measured by the trans tubular potassium gradient (TTKG; Figure 5C). Western blot analysis revealed a modest but significant increase in ROMK protein abundance in SC-51089 treated mice, corroborating the observed increase in potassium excretion and plasma K^+^ data (Figures 5A–C). Since previous studies of CA-SPAK mice compared to control showed a significant decrease in only the beta1 subunit of the BK channel, the protein abundance of only BK-beta1 was measured. There was no change in SC-51089 treated mice in protein abundance of BK channel beta1 (Figure 5E). There was also no change in either full length or cleaved forms of alpha or gamma ENaC protein abundance (Figures 6A,B). Together, these results support the conclusion that autocrine release of PGE2 from the CNT in CA-SPAK mice inhibits ROMK through EP1 receptor signaling. The role of PGE2 in mediating the specific deleterious effects of the CA-SPAK genotype on K^+^ homeostasis is uncertain, however; it is possible that PGE2-EP1 similarly controls ROMK in WT mice on the control diet.

## Discussion

In FHHt, exaggerated sodium reabsorption in the DCT influences the structure and function of the ASDN (Lalioti et al., 2006; Grimm et al., 2017), but it remains unknown if remodeling of the ASDN is driven by DCT-born paracrine factors or a chronic decrease in distal NaCl (salt) delivery. Here, we explored the involvement of PGE2 as a candidate paracrine remodeling factor, using the DCT-specific CA-SPAK mouse model to isolate the direct effects of NCC activation in the DCT from downstream consequences in the ASDN. We found constitutive activation of NCC induces PGE2 synthesis and microsomal prostaglandin E synthase-1, mPGES1, specifically in the CNT rather than the DCT, and both responses were abolished with HCTZ. Together with observations that a physiologic decrease in the apical NaCl concentration was sufficient to induce mPGES1 gene expression and increase PGE2 synthesis and release in an ASDN cell model *in vitro*, the data indicate PGE2 is activated by low distal salt delivery. Inhibition of PGE2 signaling through the EP1 receptor restored plasma potassium in CA-SPAK mice to control levels, coincident with an increase in the TTGK and a modest increase in cortical ROMK protein. Collectively the data reveal a low salt delivery activated PGE2-EP1 autocrine pathway in the ASDN that inversely couples potassium secretion in the ASDN with NCC activation.

Salt delivery to the ASDN is well-known to influence urinary potassium secretion (Khuri et al., 1975; Good and Wright, 1979, 1980; Good et al., 1984). Traditionally, this has been believed to be consequent solely to the dependence of potassium secretion on sodium reabsorption, a process called electrogenic sodium-potassium exchange. Indeed, the cellular uptake of sodium through the ENaC, creates a favorable electrical driving force for potassium to leave the cell preferentially across the apical membrane, making potassium secretion dependent on the activity of ENaC and sodium delivery. In recent years, it has become evident that sodium delivery might be physiologically regulated according to the demands of potassium balance by a potassium-sensing signaling pathway in the DCT (Terker et al., 2015; Cuevas et al., 2017; Su et al., 2019). The signaling pathway controls the activity of the thiazide-sensitive sodium-chloride co-transporter over a narrow range of extracellular potassium concentrations (Terker et al., 2015; Penton et al., 2016; Wu et al., 2018), turning off in states of potassium excess to increase distal sodium delivery, and turning-on when potassium is low to limit sodium delivery and potassium secretion. Acute changes in sodium delivery do not always change potassium excretion. Micropuncture studies by Good and Wright, revealed that potassium secretion is steeply dependent on sodium between 0 and 20 mM (K_m_ = 11), but then saturates over a more physiologic range of measured tubular fluid sodium concentrations and thus might not be sensitive to NCC mediated changes in salt delivery. Consistent with the idea that NCC-dependent sodium delivery does not always influence K^+^ secretion through electrogenic Na/K exchange. For example, Hunter et al. (2014) found that the kaliuretic response to HCTZ in mice is delayed compared to natriuresis. Our data reveal a new autocrine regulatory pathway (PGE2-EP1) that offers one mechanism to explain how sodium delivery from the DCT influences potassium secretory machinery in the ASDN, beyond electrogenic sodium potassium exchange. Activation of the system would be especially beneficial in states of chronic low extracellular potassium levels to safeguard against excessive urinary potassium loss.

We found low sodium delivery increases PGE2 synthesis by inducing the expression of mPGES1, without increasing either cyclooxygenase 1 or 2. Canonical activation of PGE2 synthesis occurs by increasing one of the cyclooxygenase enzymes, Cox1 or Cox2, in parallel with mPGES1. The inducible coupling of Cox2 and mPGES1 is well-known in inflammation, cancer and other pathophysiological settings. In the kidney, Cox2 expression is restricted to the macula densa cells, medullary interstitial cells and of the medullary collecting duct and its activity is induced by low chloride or hypertonicity (Harris and Breyer, 2001). Cox1 can also be induced with mPGES1, although this coupling mechanism is rare and usually only observed in pathological conditions compared to Cox2 (Chulada et al., 2000; Tiano et al., 2002; Okamoto et al., 2003; Akunda et al., 2004; Yu et al., 2013; Zhu et al., 2019). The ASDN is an exception to these rules; it is well-known to be a significant site of PGE2 synthesis, mediated by Cox1 and mPGES1 expression, but not of Cox2 (Komhoff et al., 1997; Campean et al., 2003) with mPGES1 as the rate limiting step of PGE2 synthesis. Our studies revealing low salt activates PGE2 by inducing mPGES1 are consistent with this. It is possible that changes in osmolarity of the tubular fluid delivered to the ASDN stimulates increased PGE2 synthesis. Our experiment was not designed to test osmolarity as a factor and instead focused on recapitulating changes in salt delivery in the native tubule that are expected in FHHt. Future studies will need to be performed to determine whether the driving factor is osmolarity or sodium or chloride.

Our studies suggest that low salt delivery induces mPGES1 gene expression. Transcriptional regulation of mPGES1 within the kidney has not been extensively studied, but is well-known to be regulated by the transcription factors EGR1 (Naraba et al., 2002; Deckmann et al., 2010), CREB (Pham et al., 2006; Deckmann et al., 2010), and KLF5 (Xia et al., 2013). RNA-seq analysis of the mouse kidney indicates mPGES1 and EGR1 are highly expressed in the ASDN, and is especially enriched in the CNT (Chen et al., 2021). Further studies are required to determine if EGR1 is indeed the activator of the PGE2-EP1 pathway in response to low salt delivery.

Although future studies are needed to test whether the results with the FHHt model can be extended to physiological circumstances of reduced distal sodium delivery, it should be pointed out the autocrine signaling system is ideally suited to translate the potassium sensing function of the DCT (Zhang et al., 2014; Terker et al., 2015; Cuevas et al., 2017; Su et al., 2020) to the potassium secretion in the ASDN. The actions of the collecting duct by PGE2 are well-known (Breyer and Ando, 1994) and our studies reinforce this notion. In isolated rat collecting ducts, PGE2 was shown to acutely inhibit ROMK activity through EP1 receptor activation in an *ex vivo* model of the rat CCD (Jin et al., 2007). We found inhibition of EP1 modestly rescues ROMK protein abundance in the renal cortex. It is possible that PGE2-EP1 mainly inhibits ROMK by inhibiting gating rather decreasing protein abundance, which would explain why potassium balance was corrected and only a modest increase of ROMK protein abundance was observed. It should be pointed out the actions of PGE2 in the ASDN are complex and depend on the predominate “EP” receptor that is expressed. For example, PGE2 was found to stimulate ENaC *via* EP4 in a mouse CCD cell line (Mansley et al., 2020). Likewise, PGE2 can stimulate AQP2-mediated water reabsorption through activation of the EP4 receptor (Gao et al., 2015; Olesen et al., 2016). In the CA-SPAK mouse model, an EP1 receptor mediated pathway was found to be involved in the inhibition of ROMK, but further studies are required to determine what controls prevailing EP receptor type in the ASDN.

The renal PGE2-EP1-ROMK signaling pathway provides one mechanism to explain the higher potassium in the CA-SPAK mouse model of FHHt, but it is not likely that it is the sole determinant of hyperkalemia in FHHt. We previously reported that CA-SPAK mice exhibit an atrophy of the CNT (Grimm et al., 2017) that is reversed by thiazide treatment, coincident with a robust rescue of ROMK and ENaC protein levels. By contrast, the EP1 antagonist did not increase ENaC protein levels, and the change in ROMK protein abundance was not nearly as profound as with thiazide. Because the tale-tell sign of structural remodeling is a large parallel change in ENaC and ROMK protein, it is not likely the EP1 antagonist restores the structure of the CNT, although laborious morphometric measurements will be required to explore if PGE2-EP1 plays a minor role in the structural remodeling response of FHHt.

It is also possible that the main effect of PGE2 on K^+^ secretion is through ENaC inhibition. In FHHt and in the CA-SPAK mice, aldosterone is maintained within normal limits, and thus it is not likely to play a role. However, EP1 also has been reported to negatively regulate ENaC channel activity (Guan et al., 1998; González et al., 2009). Since, we only measured protein abundance of ENaC, it is possible that EP1 inhibition decreased activity of the channel. PGE2 may also activate the H-K ATPase in the intercalated cell causing potassium retention. Regulation of H-K ATPase by PGE2 synthesis has been implicated in regulating gastric acid secretion (Nandi et al., 2009). Future studies will be required to ascertain if any of these processes are contributory.

Prostaglandin E2 in the kidney and urine is also increased in Bartter Syndrome, a severe salt-wasting tubulopathy caused by loss-of-function mutations in the transport molecules that are responsible for sodium-chloride absorption in the TAL and tubular fluid chloride-sensing in the macula-densa (MD). In this case, salt delivery to the ASDN is increased. At first glance, this might seem at odds with our observation that PGE2 increases in response to reduced salt delivery. However, in Bartter syndrome, PGE2 is derived from the macula densa cells (Komlosi et al., 2004). The macula densa normally increases PGE2 synthesis in response to low chloride delivery as part of a local paracrine pathway that stimulates renin release. Because luminal chloride sensing is defective in Bartter syndrome, the MD behaves as if chloride delivery is low, leading to protracted PGE2 synthesis and high levels of renin. In the DCT-specific CA-SPAK FHHt model, sodium delivery is specifically reduced downstream of the DCT and this does not stimulate renin (Grimm et al., 2017).

In summary, we found a novel inhibitory communication mechanism between NCC activity in the DCT and transport activity of ASDN segments, highlighting PGE2-EP1. The observations warrant further investigation of salt-dependent autocrine/paracrine systems, such as PGE2-EP1, as therapeutic targets for hyperkalemia that accompanies low sodium-delivery states, like CKD and heart failure.

## Data Availability Statement

The original contributions presented in the study are included in the article/supplementary material, further inquiries can be directed to the corresponding author.

## Ethics Statement

The animal study was reviewed and approved by The University of Maryland School of Medicine Institutional Animal and Care and Use Committee, and Johns Hopkins University Animal Care and Use Committee.

## Author Contributions

AZ conducted all experiments and data analysis, made figures, and wrote entire manuscript. PG contributed data for Figure 1, helped with animal dissections, and revised the manuscript. LA-Q Contributed data to Figure 6, helped with animal dissections, and revised manuscript. ED revised manuscript and engineered CASPAK mouse model. PW assisted in experimental design, figure presentation, and data interpretation, and was main editor of the entire manuscript. All authors contributed to the article and approved the submitted version.

## Funding

This work was supported in part by funds from DK054231, DK110375, DK093501, and the Foundation LeDucq.

## Conflict of Interest

The authors declare that the research was conducted in the absence of any commercial or financial relationships that could be construed as a potential conflict of interest.

## Publisher’s Note

All claims expressed in this article are solely those of the authors and do not necessarily represent those of their affiliated organizations, or those of the publisher, the editors and the reviewers. Any product that may be evaluated in this article, or claim that may be made by its manufacturer, is not guaranteed or endorsed by the publisher.
